# Antifungal activity of eco-safe nanoemulsions based on *Nigella sativa* oil against *Penicillium verrucosum* infecting maize seeds: Biochemical and physiological traits

**DOI:** 10.3389/fmicb.2022.1108733

**Published:** 2023-01-18

**Authors:** Mohamed A. Mosa, Khamis Youssef, Said F. Hamed, Ayat F. Hashim

**Affiliations:** ^1^Nanotechnology and Advanced Nano-Materials Laboratory (NANML), Plant Pathology Research Institute, Agricultural Research Center, Giza, Egypt; ^2^Plant Pathology Research Institute, Agricultural Research Center, Giza, Egypt; ^3^Agricultural and Food Research Council, Academy of Scientific Research and Technology, Cairo, Egypt; ^4^Department of Fats and Oils, Food Industries and Nutrition Research Institute, National Research Centre, Giza, Egypt

**Keywords:** *Nigella sativa* oil, nanoemulsions, antifungal, maize seeds, *Penicillium verrucosum*

## Abstract

The main goals of the present investigation were to develop O/W nanoemulsion fungicides based on cold-pressed *Nigella sativa* (black seed) oil to prevent *Penicillium verrucosum* infection of maize seeds and to test their antifungal activity against this fungus. Additionally, the effect of these nanoemulsions on plant physiological parameters was also investigated. Two nonionic surfactants namely Tween 20 and Tween 80 were used as emulsifying agents in these formulations. The effect of sonication time and surfactant type on the mean droplet size, polydispersity index (PDI), and zeta potential of the nanoemulsions were determined by dynamic light scattering (DLS). Results indicated that both sonication time and emulsifier type had pronounced effects on the stability of O/W nanoemulsions with a small particle size range (168.6–345.3 nm), acceptable PDI (0.181–0.353), and high zeta potential (−27.24 to –48.82 mV). Tween 20 showed superior stability compared to Tween 80 nanoemulsions. The *in vitro* results showed that complete inhibition of *P. verrucosum*-growth was obtained by 10_T80 and 10_T20 nanoemulsions at 100% concentration. All nanoemulsions had increment effects on maize seed germination by 101% in the case of 10_T20 and 10_T80 compared to untreated seeds or the chemical fungicide treatment. Nanoemulsions (10_T20 and 10_T80) were able to stimulate root and shoot length as compared to the fungicide treatment. Seed treatment with 10_T80 nanoemulsion showed the highest AI and protease activity by 75 and 70%, respectively, as compared to the infected control. The produced nanoemulsions might provide an effective protectant coating layer for the stored maize seeds.

## 1. Introduction

Maize (*Zea mays* L.) is the most widely grown and consumed grain in the world (Shahbandeh, 2022) with a production of 1,162,352,997 tons, and a harvested area of 20,198,3,645 ha. In 2020, Egypt produces 7,500,000 tons of maize from the harvested area of 1,458,881 ha (FAOSTAT, 2022). The Food and Agriculture Organization of the United Nations predicts that the world target of 2.4% annual production yield development will not be met, with estimates predicting a 7% yield decline due to biotic and abiotic factors (Admin MINADER—Assessment of the 2019/2020 Crop Year and Food Availability in the Adamawa, East, Far, 2022; Dinango et al., 2022). Overall, food and feeds such as cereals are commonly contaminated with mycotoxigenic fungi and mycotoxins, which are a serious problem faced by many countries (Lee and Ryu, 2017). This causes significant losses to producer countries when their exports are rejected because they do not meet the legislative limits. The Rapid Alert System for Food and Feed shows that up to 30% of commodities imported into the EU are rejected because of mycotoxin contamination (Rapid Alert System for Food and Feed, 2018). Fungal species from the genera Penicillium, Fusarium, Alternaria, Claviceps, Stachybotrys, and Aspergillus are of greatest concern in terms of mycotoxin contamination of food and feed (Milićević et al., 2010). In particular, *Penicillium verrucosum* is a fungal pathogen that commonly affects grain crops produces citrinin (CIT), ochratoxin A (OTA), patulin (PAT), and penicillic acid (PA) in storage (Frisvad et al., 2004, 2005; Limay-Rios et al., 2017).

Synthetic fungicides are considered the most effective solution against a wide range of fungal diseases. However, their long-term use has induced undesirable pathogen resistance. Besides, their residues in soil and food are detrimental to human beings and the environment (Mustafa and Hussein, 2020; Alghuthaymi et al., 2021). Thus, scientists are engaged in the development of alternative green eco-safe antifungal agents such as plant-based or specialty vegetable oils nanomaterials (Alam et al., 2014). In this context, innovations in nanoemulsion-based agrochemicals could present great enhancement in active ingredient solubility, improving agrochemical bioavailability, wettability, and stability properties during the application, and resulting in better efficiency for integrated disease/pest management.

Several alternative control means were proposed to manage plant diseases and their causal agents. Black cumin (*Nigella sativa*), also known as al-habba al-sawdaa, or senouj, is a member of the botanical family of Ranunculaceae. Nigella sativa seeds are high in unsaturated and essential fatty acids and contain about 28–36% fixed oil. According to numerous studies, volatile oil concentration can range from 0.4 to 2.5% (Nickavar et al., 2003; Sultan et al., 2009; Rahim et al., 2022).

Previously, El-Nagerabia et al. (2012) assessed the inhibitory effect of *N. sativa* oil and *Hibiscus sabdariffa* calyx extract on the growth and aflatoxin B1 production by *Aspergillus parasiticus* and *Aspergillus flavus* strains at various doses. Also, Thoppil et al. (1998) studied the activity of *N. sativa* against *Fusarium semitectum, Aspergillus niger*, and *Rhizopus stolonifer*. Soil drenching with black seed oil was found to be highly effective against mycelial growth and conidia sporulation of *Fusarium* spp. and significantly reduced root rot incidence of grapevine plants (Ziedan et al., 2020). Essential oils are considered safe eco-friendly secondary metabolites of higher plants containing about 60 bioactive components of terpenes, alcohol, phenols, aldehydes, and esters (Bakkali et al., 2008), which are compatible with biotic and abiotic agents in controlling several plant diseases including foliar, wilt, root rot, and postharvest fungal decay (Abbasi et al., 2003; Kadoglidou et al., 2020; Ziedan et al., 2020).

Nanoemulsions formulations of clove and black seed essential oils were applied as a soaking treatment of cucumber fruit for management of postharvest spoilage caused by *Galactomyces candidum, Alternaria tenuissima,* and *Fusarium solani* (Mossa et al., 2021). In addition, Ziedan et al. (2022) summarized that foliar application of nanoemulsion formulation of clove and black seeds is more promising than fungicides in controlling gray mold on cucumber fruits caused by *Botrytis cinerea* in plastic greenhouses with no phytotoxicity on cucumber plants.

To overcome the above-mentioned challenges especially those related to synthetic fungicides and the obstacles associated with climate change, it is urgently necessary to develop alternative eco-safe methods. Therefore, the main objectives of the current study were (i) to produce an eco-safe, green *N. sativa*-based O/W nanoemulsion fungicide to protect maize seeds from *P. verrucosum* infection; (ii) to investigate the influence of surfactant type and sonication time on the formation of nanoemulsions using dynamic light scattering (DLS); and (iii) to test the antifungal activity of the developed nanoemulsions against *P. verrucosum in vitro* and study their effect on seed germination, maize plant growth, and physiological parameters.

## 2. Materials and methods

### 2.1. Materials

Cold pressed*-N. sativa* oil was purchased from the oil extraction unit, National Research Center, Egypt. Tween 20 [Polyoxyethylene (20) sorbitan monolaurate], Tween 80 [Polyoxyethylene (20) sorbitan monooleate], Streptomycin sulfate salt, Tris–HCl buffer, and bovine serum albumin (BSA) were purchased from Sigma Aldrich, United States. Potato dextrose agar (PDA) was purchased from Merck KGaA, Darmstadt, Germany. Sodium hypochlorite solution 4–6% was provided by Loba Chemie Pvt. Ltd. India.

### 2.2. Physico-chemical characterization of *Nigella sativa* oil

According to the American Oil Chemists Society’s (AOCS) standard procedures, the peroxide value (PV), acid value (AV), saponification value (SV), and iodine value (IV) were assessed (American Oil Chemists' Society, 2005; American Oil Chemists' Society, 2011). Fatty acid methyl esters of *N. sativa* oil were prepared using a modified trans-methylation method according to AOAC (2016), Official Methods of Analysis 2017. Determination of fatty acids composition was carried out using a Hewlett Packard HP 6890 gas chromatograph, operated under the following conditions: Detector, flame ionization (FID); column, capillary, 30.0 m × 530 μm, 1.0 μm thickness, polyethylene glycol phase (INNO Wax); N2 with flow rate, 15 ml/min with average velocity 89 cm/s (8.2 psi); H2 flow rate, 30 ml/min; air flow rate, 300 ml/min; split ratio, 8:1, split flow, 120 ml/min; gas saver, 20 ml/min. Detector temperature, 280°C; column temperature, 240°C; and injection temperature, 280°C. Programmed temperature starting from 100°C to reach a maximum of 240°C was used for eluting the fatty acid methyl esters. The identification of the peaks was made as compared with chromatograms of authentic standard fatty acids methyl esters mixture purchased from Sigma (United States).

### 2.3. Preparation of *Nigella sativa* oil nanoemulsions

Tween 20 (HLB = 16.7) and Tween 80 (HLB = 15) are non-ionic surfactants that are widely used as emulsifiers and stabilizers in pharmaceutical formulations, as well as in the food and cosmetic industries. Preparation of the coarse and subsequently the nanoemulsion was carried out according to Hashim et al. (2019) with some modifications. In brief, 2% tween 20 or 80 surfactants were mixed with *N. sativa* oil (10%) as the dispersed oily phase at 600 rpm for 5 min with a magnetic stirrer. The prepared oily phase (12% by weight) was then added to the distilled water (aqueous phase, 88%) while being continuously stirred magnetically for 15 min. To develop the coarse emulsions, this pre-emulsion was further homogenized for 3 min at 16.000 rpm using a high-speed homogenizer (X520, CAT Ingenieurburo M. Zipperer, GmbH, Germany).

**Figure 1:**
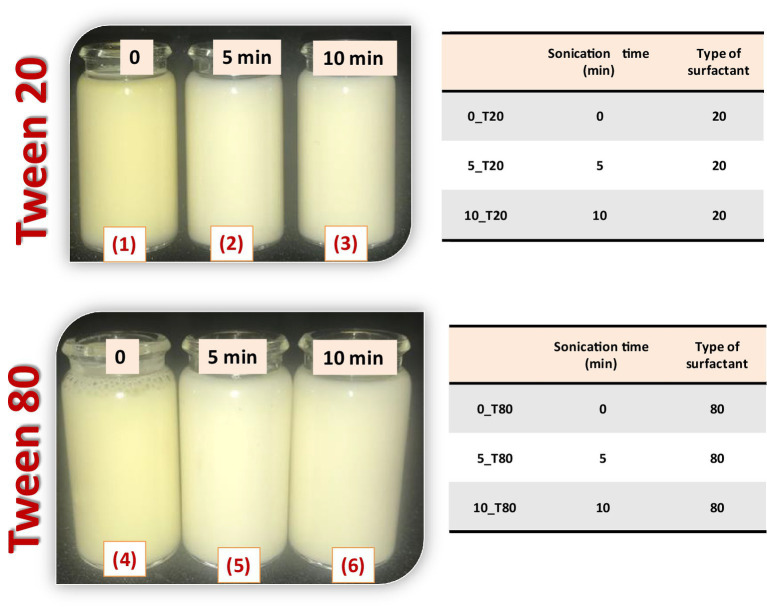
Preparation of *Nigella sativa* oil nanoemulsions using two types of surfactant (Tween 20 and 80) and different times of sonication (0, 5, and 10 min).

An ultrasonication process was used to produce nanoemulsions. The previously prepared coarse emulsions were sonicated using a probe sonicator (Sonics & Materials Inc., 53 Church Hill Rd., Newtown, CT, United States) with a diameter of 22.5 mm at 60% of full power amplitude (60 W) to achieve homogenous nanoemulsions. To evaluate the effect of the sonication times on coarse emulsions, different times of sonication (0, 5, and 10 min) were used (Figure 1). All samples were made at room temperature (25 ± 2°C). To evaluate the effect of surfactant type on the preparation, droplet size, and stability of *N. sativa* nanoemulsions, Tween 20 and Tween 80 were used under the same conditions. Finally, the constituent of the prepared nanoemulsions are quantitatively the same, but differ from one to another in the type of the surfactants and sonication time (0_T20, 5_T20, 10_T20; 0_T80, 5_T80, and 10_T80).

### 2.4. Characterization of nanoemulsions

To study emulsion stability, 10 ml aliquots of each sample were immediately transferred to 10 ml graduated cylinders, sealed, and kept at room temperature for 7 days. Then, the volume of the upper phase was measured. The stability was calculated as a percentage of separation and expressed as:


$$\mathrm{Separation}\;\left(\%\right)=H_{1}/H_{0}\times 100$$

where *H_o_* represents the initial height of the emulsion and *H_1_* is the upper phase height (Carneiro et al., 2013).

The viscosity of nanoemulsions was also measured at room temperature using a Brookfield digital viscometer (Middleboro, MA, United States). The samples were put in a glass cylinder and the spindle (S^−1^) was rotated at different speeds, ranging from 20 to 100 rpm. Measurements were repeated at least three times for each rpm. The viscosity was expressed as centipoise (cP).

The mean droplet size, size distribution, and zeta potential of the nanoemulsions were determined by dynamic light scattering using a ZetasizerNano ZS (Malvern, United Kingdom) at 30°C. Magnetic agitation was used to suspend 30 μl of each nanoemulsion in 970 μl water. The droplet size distribution was observed throughout each measurement until the measurements were constant. Every experiment was run in triplicates.

To examine the morphology of the prepared samples, Transmission electron microscopy HR-TEM (JEOL, JEM-2100, Tokyo, Japan), operated at 200 kV was used. The prepared nanoemulsions were applied to a copper grid, covered with carbon (carrier powder), allowed to air dry then investigated.

### 2.5. Fungal pathogen identification and culture conditions

Stored maize seeds infected with the pathogenic fungus *P. verrucosum* producing Ochratoxin A (OTA) were collected during 2022 season. *P. verrucosum* was isolated and purified from infected seeds and cultivated onto potato dextrose agar (PDA) medium, amended with streptomycin sulfate (300 mg/L). Fungal cultures were incubated for 14 days at 26°C. *Penicillium verrucosum* was firstly identified using a morphological identification key (Nelson et al., 1983; Hirano et al., 2006).

The molecular identification was performed based on the 5.8S nuclear ribosomal gene. Around 100 mg of harvested mycelium was ground to a fine powder in the presence of liquid nitrogen. The ground mycelium was transferred to a 1.5 ml microcentrifuge tube and DNA was extracted using the DNeasy plant mini kit (Qiagen, Hilden, Germany), according to the manufacturer’s recommendations. The PCR mixture and thermal conditions were performed according to Çolak and Biçici (2013). PCR amplification and sequencing of the internal transcribed spacer (ITS) region of rDNA were performed to molecular identify *P. verrucosum* using the ITS1 and ITS4 primers (White et al., 1990). PCR reactions were performed in a 25 μl final mixture volume containing 2 μl of 10 ng/μl of genomic DNA, 2.5 μl of 10× PCR buffer, 1 μl of dNTPs 10 mM each, 1.5 μl of 25 mM MgCl_2_, 0.5 μl each of forward and reverse primers (0.5 mM), and 0.2 μl of Taq DNA polymerase (5 U/μl; Biomatik LLC, Canada). The amplification program included an initial denaturalization cycle of 3 min at 94°C, followed by 35 cycles of 15 s at 95°C, 30 s at 53°C, 80 s at 72°C, and a final extension step of 10 min at 72°C, in a thermal cycler (Applied Biosystems, United States). After amplification, the PCR products were analyzed by electrophoresis in a 1% agarose gel. PCR products were purified and sequenced using the same forward and reverse primers. ABI trace files were analyzed and contigs were constructed using the BioEdit sequence alignment editor. Sequences of the fungal isolate were deposited in the GenBank.

### 2.6. *In vitro* experiments

#### 2.6.1. Antifungal activity of the nanoemulsions

The antifungal activity of the prepared nanoemulsions namely0_T20, 5_T20, 10_T20, 0_T80, 5_T80, and 10_T80 against *P. verrucosum* was determined at three concentrations (25, 50, and 100%) following the growth rate method. Briefly, *P. verrucosum* mycelial disks (7 mm in diameter) developed on PDA plates were carefully cut from the colony’s plate edges and deposited on plates supplemented with 1 ml of the above-mentioned concentrations of the nanoemulsions. The inoculated plates were then incubated at 25°C ± 1 for 4, 6, and 8 days. Untreated plates served as a control. Mycelial fungal radial growth was monitored after incubation in all plates, and data were reported as the percentage of inhibition (Çolak and Biçici, 2013):


Inhibition rate of fungal growth(%)=[(A−a)/A]×100

Where A represents the fungal mycelial radial growth on the control (untreated) plate and a represents the fungal mycelial radial growth on the plate treated with three concentrations of each nanoemulsion individually. All laboratory experiments were repeated twice in triplicates under controlled conditions.

#### 2.6.2. Inoculum preparation of *Penicillium verrucosum* (nano-seed priming and protection activity of nanoemulsions)

Maize seeds (cv. Boushy) were sterilized with an aqueous solution of sodium hypochlorite (0.25%, v/v) for 3 min. Seeds were then copiously washed with distilled water. To prime seeds, seeds were immersed in 10 ml of each of the prepared nanoemulsions (100% concentration) individually and maintained for 4 h under slow stirring. Afterward, seeds were placed on sterilized filter papers in Petri dishes (4–5 seeds/plate), supplemented with 5 ml of distilled water and 1 ml of (1 × 10^6^ spore) *P. verrucosum* spore suspension. The inoculated plates were then incubated in the dark (24 ± 2°C). Untreated seeds, infected seeds, and the chemical fungicide treated-seeds were used as controls. The percentages of germinated seeds and their radical length were recorded in comparison to control plates at 5 days after priming.

### 2.7. Physiological studies

#### 2.7.1. Plant growth, treatments, germination, shoot, and root length

Seeds of maize (cv. Boushy) were sterilized as mentioned above. Seeds were then cleaned multiple times with distilled water. To prime maize seedlings, five nanoemulsions with the most appreciated concentration (100%) resulted in antifungal activity experiments at the laboratory were created. The seeds were subsequently dipped in 10 ml of these solutions for 6 h in gentle agitation for seed priming. The seeds were then placed on sterilized filter paper in Petri plates (4–5 seeds per plate) and 10 ml of distilled water was added. These samples were covered and put in a dark growing room at 22 ± 2°C. Seeds for controls were collected to prime them with distilled water. The percentage of seed germination and radical lengths were measured 4 and 5 days post-sowing (DPS), respectively. Untreated control, chemical fungicide, and infected control were used as controls.

#### 2.7.2. Determination of chlorophyll, carotenoids, anthocyanin, and soluble protein

At the third leaf stage (14 DPS), plants were collected and the physiological effects of the tested nanoemulsions on plant height, dry weight, and the level of some photosynthetic pigments (chlorophyll and carotenoid content) were evaluated in all treated seed samples. The content of photosynthetic pigments was assessed according to Venkatachalam et al. (2017). In this regard, 100 mg of maize fresh leaves were collected and grounded with 5 ml of 80% (v/v) ice-cold acetone using mortar and pestle and homogenate was aliquoted into sterile centrifuge tubes and centrifuged at 6,000 rpm for 6 min. The supernatant was then transferred into fresh centrifuge tubes and the pellet was re-extracted with 2.5 ml of 80% (v/v) ice-cold acetone, and it was repeated twice. The level of Chlorophyll a, b, and carotenoid contents was quantified using a double beam UV–Visible spectrophotometer (Spectrophotometer UV-1800, Shimadzu Tokyo, Japan) at wavelengths of 663, 645, and 470 nm, respectively. Determination of chlorophyll a, b, and carotenoid contents level was carried out using the equations proposed by Lichtenthaler (1987) and expressed as mg g^1^ FW.


$$Chla=12.25\times A_{663}-2.79\times A_{645}$$

Chlb=21.50×A645−5.10×A663

Car=(1,000×A470−1.82×Chla−85.02×Chlb)/198

While anthocyanin content was determined according to the protocol of Lichtenthaler and Buschmann (2001), the anthocyanin concentration was evaluated spectrophotometrically at 535 and 650 nm.

For protein assays, a cold mortar and pestle were used to homogenize around 50 mg of leaf tissues in 5 ml of 0.1 M Tris–HCl buffer (pH 7.5). At 4°C, the extract was centrifuged for 15 min at 10,000 rpm. The protein content of the supernatant was then calculated using Bradford’s method (Bradford, 1976) using a standard of bovine serum albumin (BSA). Untreated control, chemical fungicide, and infected control were used as controls.

#### 2.7.3. Determination of acid invertase and protease activities

Acid invertase (AI) activity of leaves was assayed according to Hwang and Heitefuss (1986). Following pathogen inoculation, 0.25 g of leaf segments were collected from both the control and infected leaf tissues at the third leaf stage (14 DPS), submerged in ice-cold ethyl acetate for 20 min, and then rinsed in ice-cold distilled water. Each sample was kept in a water bath at 30°C for 60 min, during which time it was exposed to 0.1 M sodium phosphate buffer (pH 5.6), 0.5 M sucrose, and distilled water. A piece of filter paper was used to soak up any remaining moisture from the leaf surface. Twenty milliliter of 0.5 M sucrose, 2 ml of 0.1 M sodium phosphate buffer (pH. 5.6), and 6 ml of double-distilled water were placed in a 20 ml vial for each leaf sample. In a water bath with shaking, the vials were kept at 30°C for 60 min. AI activity of all samples was measured at 280 nm in a spectrophotometer (Hitachi Model: U-1100 573 × 415). Also, protease activity was assessed following the method of McDonald and Chen (1965). In this regard, about 100 mg of leaf tissue was incubated at 30°C for 1 h in 4 ml of a substrate solution (1% casein in 0.1 M sodium citrate buffer, pH 7.0). The remaining protein was precipitated by adding 5 ml of trichloroacetic acid (TCA) at a concentration of 5%. After 30 min, the tubes’ contents were filtered through filter paper to remove the precipitate (WHATMAN No. 40). After filtration, a 5-ml aliquot of the filtrate was combined with a 1-ml aliquot of an alkaline reagent mixture made from 2% sodium carbonate, 2.7% sodium potassium tartrate, and 1% copper sulfate. We then made the solution alkaline by adding 2 ml of 1 M sodium hydroxide. The Folin–Ciocalteu-phenol reagent (0.5 ml) was added after the mixture had been allowed to stand for at least 10 min. After 30 min, a spectrophotometer was used to determine the absorbance of the blue color at 660 nm in each sample. One unit of protease activity was defined as the amount of enzyme required to produce an increase in optical density at 660 nm of 0.1 h^−1^ at 30°C at pH 7.0. Untreated control, chemical fungicide, and infected control were used as controls.

#### 2.7.4. Estimation of polyphenol oxidase activity

Polyphenol oxidase (PPO) activity was determined following the method of Kar and Mishra (1976) with minor modifications. Leaf tissue (1 g) collected at the third leaf stage (14 DPS) was homogenized in 2 ml of 0.1 M sodium phosphate buffer (pH 6.5) and centrifuged at 10,000 × *g* for 25 min at 4°C. Three milliliter reaction mixture contained 25 mM phosphate buffer (pH 6.8), 0.1 Mm pyrogallol, and 0.1 ml enzyme extract. The control reaction mixture contained no pyrogallol. The absorbance of each sample was recorded at 420 nm.

#### 2.7.5. Estimation of peroxidase and chitinase activities

Leaf tissue (1 g) collected at the third leaf stage (14 DPS) was homogenized in 5 ml of 0.05 M phosphate buffer (pH 7.0) containing 10% polyvinylpyrrolidone (PVP-SIGMA) and 0.1 M ethylene di-amine-tetra-acetic acid (EDTA-SIGMA) and centrifuged at 14,000 rpm for 20 min at 4°C. POD activity was measured by the method of Abo-Elyousr et al. (2020). The assay mixture contained 0.1 ml enzyme extract, 1.35 ml 0.1 M mM MES buffer (pH 5.5), 0.05% H_2_O_2_, and 0.1% phenylenediamine. Changes in the absorbance in each sample were recorded at 485 nm for 3 min. Chitinase activity was determined by colorimetric assay following the protocol of Stangarlin and Pascholati (2000).

In this regard, the substrate CM-chitin-RBV (200 μl of 2 mg/ml) was gently mixed with 300 μl of leaf protein extract and 250 μl of 10 mM Tris–HCl, pH 7.5, containing 1% Triton X-100. The mixture was incubated at 37°C for 3–4 h. The reaction was stopped by the addition of 200 μl of 2 M HCl. Samples were then cooled on ice for 20 min followed by centrifugation at 20,000 × *g* for 10 min to remove the non-degraded substrate. The supernatant was collected and one unit of chitinase activity represented an increase in absorbance of 0.1 at 550 nm (Lopes et al., 2008). Untreated control, chemical fungicide, and infected control were used as controls.

### 2.8. Scanning electron microscope

*Penicillium verrucosum* fungal cultures grown on PDA treated or not with 10_Tween 20 or 10_Tween 80 for 1 week were subjected to a scanning electron microscopy (SEM) examination at the National Research Center (NRC), Central Unit for Scientific Analysis and Services in Giza, Egypt to study their possible mode of action against *P. verrucosum* following the protocol of Youssef et al. (2019). Briefly, 5 × 10 mm fungal blocks were carefully cut from a culture grown on PDA plates and immediately inserted in bottles containing 3% glutaraldehyde in 0.05 M phosphate buffer (pH 6.8) at 4°C. The prepared samples were stored in this solution for 48 h to be repaired and then washed 3–4 times for 20–25 min each with distilled water. The fungal samples were then dehydrated for 20 min in the ethanol series dilution and then dried in liquid carbon dioxide. The prepared samples were fixed using standard double-sided adhesive on standard ½-inch SEM nozzles and with gold–palladium galvanoplastic (60 s, 1.8 mA, 2.4 kV) in the coated Poutron SEM Coat coating system. All samples were finally subjected to examination in JEOL JXA-480 SEM (JEOL, Tokyo, Japan), which operated at 15 kV at 6000-fold magnification.

### 2.9. Statistical analysis

Statistical analysis was performed for significant variations by using R software packages (version 4.0.5; R Core Team, 2019). All analyses were repeated twice, performed in triplicate, and expressed as mean ± SD. Data were subjected to a one-way ANOVA. Duncan’s multiple range test (*p* ≤ 0.05) was used to distinguish the differences among various treatments and the error bars were plotted in respective figures.

## 3. Result and discussion

### 3.1. Fatty acids composition and physicochemical characteristics of *Nigella sativa* oil

**Figure 2:**
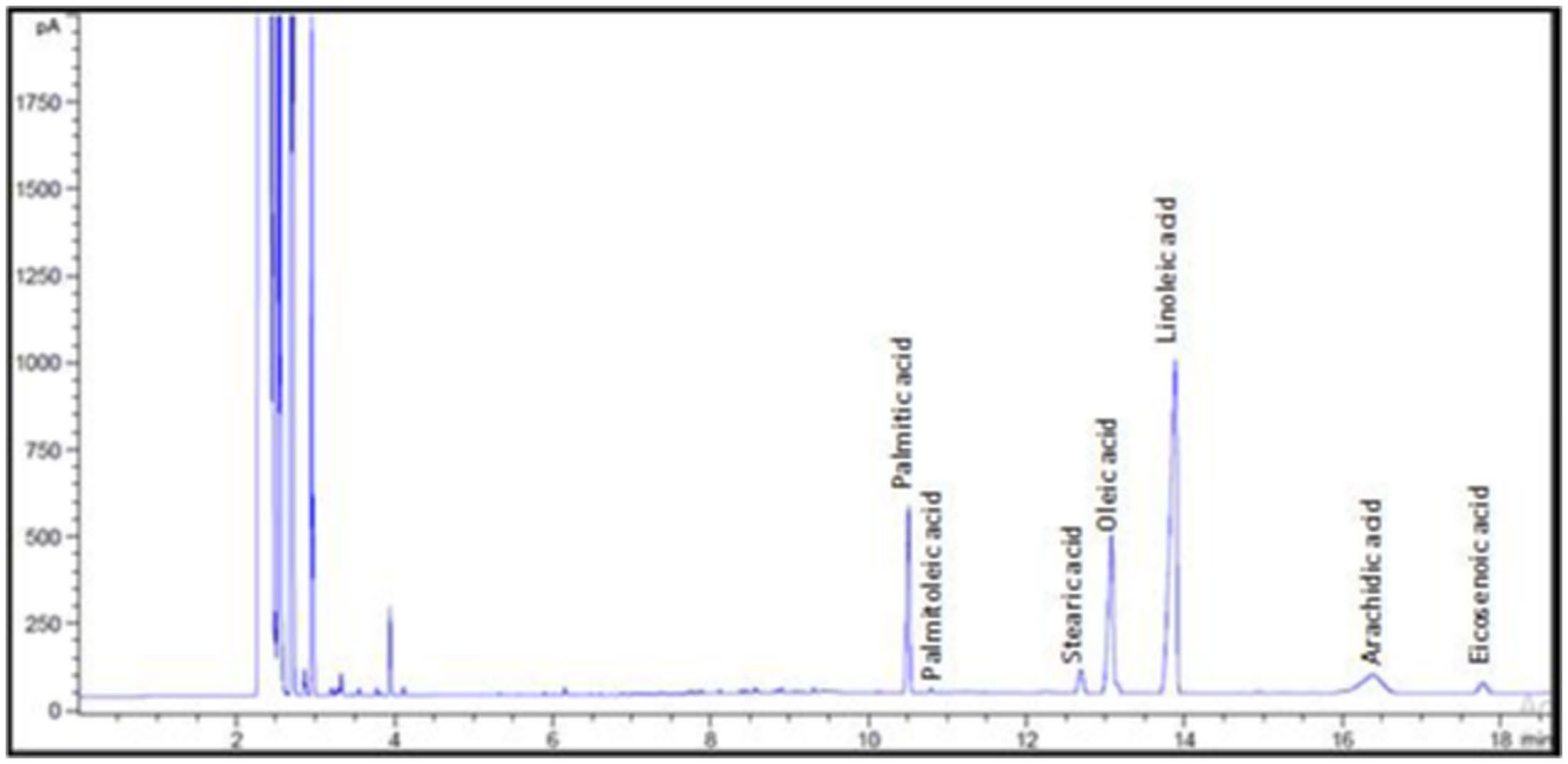
Fatty acid composition of *Nigella sativa* oil.

According to the analysis of the fatty acid composition of investigated oil (Figure 2), linoleic, oleic, and palmitic acids constitute more than 84.50% of the total fatty acids in *N. sativa* oil. Linoleic acid (18:2n-6) composed 51.81% of the total fatty acids in the sample, followed by oleic acid (18:1n-9) at 19.77/100 g and palmitic acid (16:0) at 13.01/100 g. The percentages of saturated fatty acids (SFA) and unsaturated fatty acids (UFA) were 74.15 and 25.85%, respectively. These findings support earlier studies that found linoleic acid to be the most prevalent polyunsaturated fatty acid (PUFA) in *N. sativa* (Lutterodt et al., 2010). *Nigella sativa* oil was found to have a peroxide value of 62.26 meq.O_2_/kg oil and an AV of 14.31 mg KOH/g. According to Goerlich Pharma International (2007), *N. Sativa* seeds oil peroxide value is rising rapidly immediately after production as a result of the residues of essential mock oxygen of the fatty oil bound as peroxide. The peroxide and the acid values of *N. sativa* seed oil from Egypt were found to be <120 meq O_2_/kg oil and <20 mg KOH/g oil, respectively (Goerlich Pharma International, 2007). The investigated oil had an IV of 122.7 mg/100 g. An increased level of unsaturation is indicated by a higher iodine value (Marina et al., 2009). The oil under investigation had a saponification value of 188.9 mg/100 g. It is possible to determine the kind of triglycerides in a sample by using the saponification value, a measurement of the alkali-reactive groups in oils. High SV values indicate high molecular weight fatty acids content in the sample (Haile et al., 2019). The two values (IV and SV) measure the molecular weight and the number of double bonds in the triglyceride molecule, respectively.

### 3.2. Effect of sonication time and surfactant type

The type of homogenizer utilized, production temperature, energy intensity, and time, as well as the state of the sample in the form of oil, oil concentration, type of emulsifier/surfactant employed, and the physicochemical characteristics of the sample, all influence the droplet size and zeta potential (Tian et al., 2022). The results showed that both tested surfactants (Tween 20 or Tween 80) can form a stable nanoemulsion under experimental conditions. However, sonication time and surfactant type have a significant influence on droplet size and zeta potential of the prepared nanoemulsions.

#### 3.2.1. Droplet size of nanoemulsions

**Figure 3:**
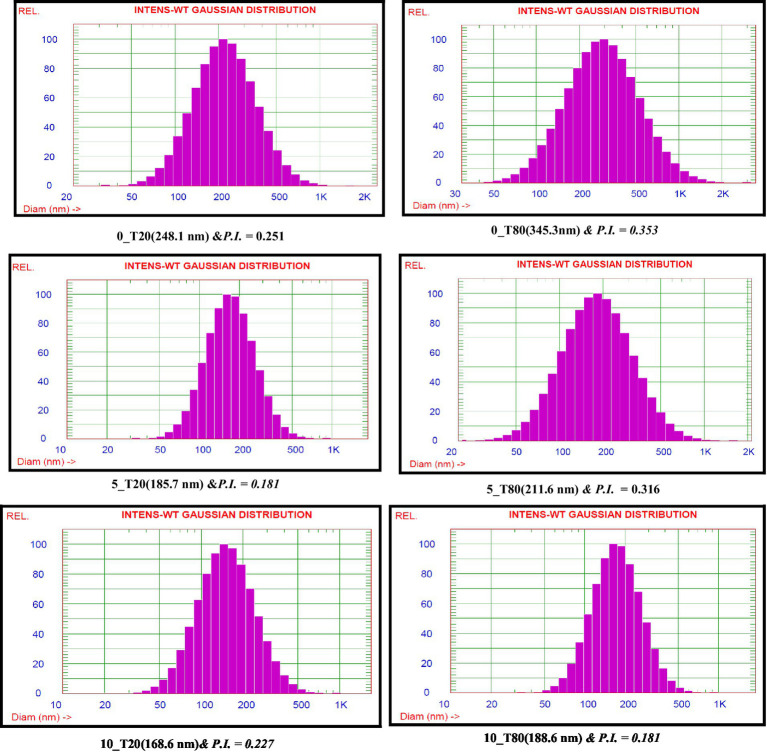
Nanoemulsion droplet size distributions of *Nigella sativa* oil using two surfactants and different sonication times.

Emulsifiers are required in the synthesis of nanoemulsions because they lower interfacial tension and make it easier for small droplets to form during homogenization *via* oil–water interface adsorption (Leão et al., 2019). The time of the ultrasonication was found to have a great impact on the droplet size of the resulting nanoemulsions formulated with either Tween 20 or Tween 80. In this study, the droplet size of 0_T20, 5_T20, and 10_T20 nanoemulsions were 248.1, 185.7, and 168.6 nm, respectively. While, the droplet size of 0_T80, 5_T80, and 10_T80nanoemulsions was 345.3, 211.6, and 188.6 nm, respectively (Figure 3). Figure 3 depicts the average size of nanoemulsions produced using the ultrasonication technique at various ultrasonication times. The results of this study showed that droplet size was greatly influenced by the time of the sonication (0, 5, and 10 min) and the longest sonication time revealing the smallest droplets for both emulsifiers (Figure 3). A longer sonication time causes more shear forces to be applied to the droplets, which leads to greater droplet deformation and fragmentation, and thence, smaller droplet sizes are expected. Results indicated that Tween 20 which has a shorter chain fatty acid moiety (lauric acid, 12 carbon atom) caused lower droplet size nanoemulsion compared to Tween 80 which has longer chain fatty acid namely oleic acid (18 carbon atom chain). The effect of the emulsifier type was also demonstrated by other researchers studies (Chong et al., 2018; Maccelli et al., 2020; Mohammed et al., 2020; Syapitri et al., 2022) who found that the type of emulsifier and homogenization time influenced droplet size and stability of prepared nanoemulsions.

It is well known that the particle size distribution index or polydispersity index (PDI) of nanoemulsion droplets is an essential factor for investigating the stability of nanoformulations in terms of physical analysis and nanoemulsion stability (Kurpiers et al., 2020). The homogeneity of nanoemulsions was assessed using the PDI, which ranged from 0.1 to 1.0. In this study, PDI values ranged from 0.181 to 0.353 (Figure 3). Nanoemulsions with lower PDI values are thought to be more homogenous. Also, an acceptable level of homogeneity is indicated by nanoemulsions with a PDI of less than 0.6 (Eid et al., 2013).

#### 3.2.2. Zeta potential and emulsion stability

**Figure 4:**
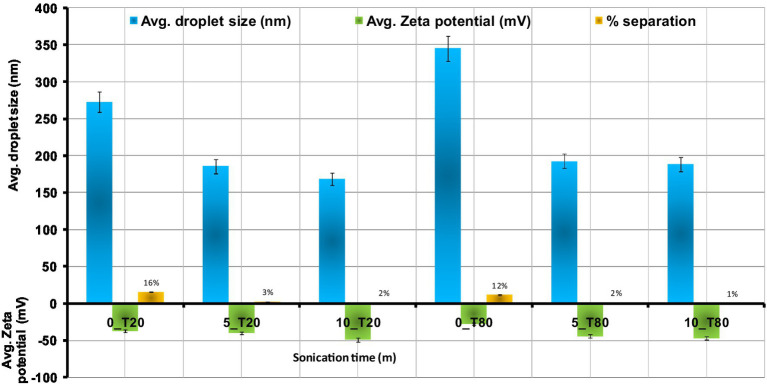
Effect of sonication time on the droplets size, zeta potential, and separation % of *Nigella sativa* oil nanoemulsions using ultra-sonication method means ± SD (*n* = 3).

The Zeta potentials of the nanoemulsions prepared using Tween 20 and Tween 80 are displayed in Figure 4. As shown in the Figure 4, the highest zeta potential values for the synthesized nanoemulsions using Tween 20 and Tween 80 were obtained after 10 min of sonication. It was found that Zeta potential was increased by increasing the sonication time. The obtained data show that the zeta potential of the Tween 20 nanoemulsions is highest at 10 min (−46.74 mV), followed by 5 min (−39.67 mV), and 0 min (−37.34 mV). Tween 80 nanoemulsions showed the same trend with decreasing sonication time, where zeta potential of 0, 5, and 10 min recorded –27.24, −44.12, and −48.82 mV, respectively. These results may be explained by the fact that oil droplets with smaller surface areas have larger surface charges. Nanoemulsions could have a greater negative zeta potential and become more stable due to the higher surface area and smaller particle size. The obtained results were in line with the findings of Qushawy et al. (2022). The surface charge density of the produced nanodroplets, or zeta potential, is directly related to the stability of the electrostatic field and the tendency of the nanodroplets to aggregate (Ziani et al., 2011; Chung et al., 2017; Jarzębski et al., 2021). Theoretically, a high zeta potential value indicates good stability. In general, it is considered that zeta potential values above the range of –30 to +30 mV have enough repulsive force to achieve greater physical colloidal stability. Furthermore, the formation of a hydrogen bond between the water molecules and the oxyethylene group of Tweens may cause the hydroxyl ion to be selectively adsorbed at the oil/water interface, producing a negative charge (Liu et al., 2006). Visual observation of the nanoemulsions at regular intervals during 7 days indicated that 0_T20 and 0_T80 nanoemulsions showed the highest separation percentages of about 16 and 12%, respectively. While 5_T20 10_T20, 5_T80, and 10_T80 displayed physical stability against gravitational separation with slight oil separation of about 1–3% (Figure 4).

#### 3.2.3. Viscosity determination

**Figure 5:**
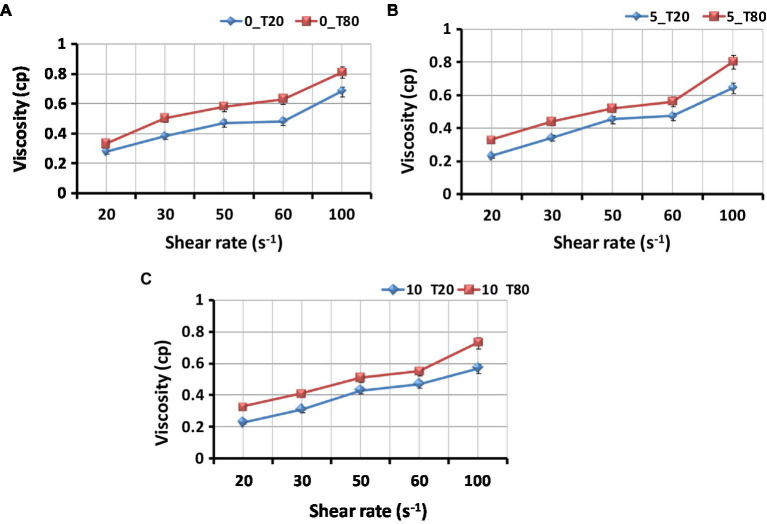
Effect of type of surfactant on the viscosity of the *Nigella sativa* oil nanoemulsions. **(A)** 0_T20 and 0_T80; **(B)** 5_T20 and 5_T80; **(C)** 10_T20 and 10_T80; Bars represent standard error of means (SEM).

Figure 5 shows the viscosities of the nanoemulsion produced with the different emulsifiers at different sonication times. The nanoemulsion viscosity depends on the oil, water, and emulsifier components. The compositional and structural effects of interfacial packing (the ratio of tail-to-head cross-section) are also related to the viscosity change with water content. In the case of Tween 80 compared to Tween 20, the rise in nanoemulsion viscosity measurement is more noticeable (Figure 5). The lowest viscosity was found in sample 10_T20, whereas sample 0_T80 had the highest viscosity. The low viscosity may be the result of a high water content combined with a particular type of emulsifier. Tween surfactants often contain a variety of hydrophobic groups. Tween 80 has longer side chains that are responsible for more viscosity than Tween 20. Tween 20 reduced the interfacial tension and viscosity between water and oil (Taha et al., 2020). Increased sonication time resulted in a nanoemulsion with a reduced viscosity, which will increase its stability. In the same context, Gorjian et al. (2022) studied the viscosity of spearmint essential oil nanoemulsions with various surfactants (Tween 20, Tween 40, and Tween 80). An increase in hydrogen bonding causes a reduction in the molecular distances of emulsion systems and an increase in flow resistance, which means an increase in the viscosity of the system. Thus, the stability of nanoemulsions is greatly influenced by their viscosity.

### 3.3. Morphology of nanoemulsions

**Figure 6:**
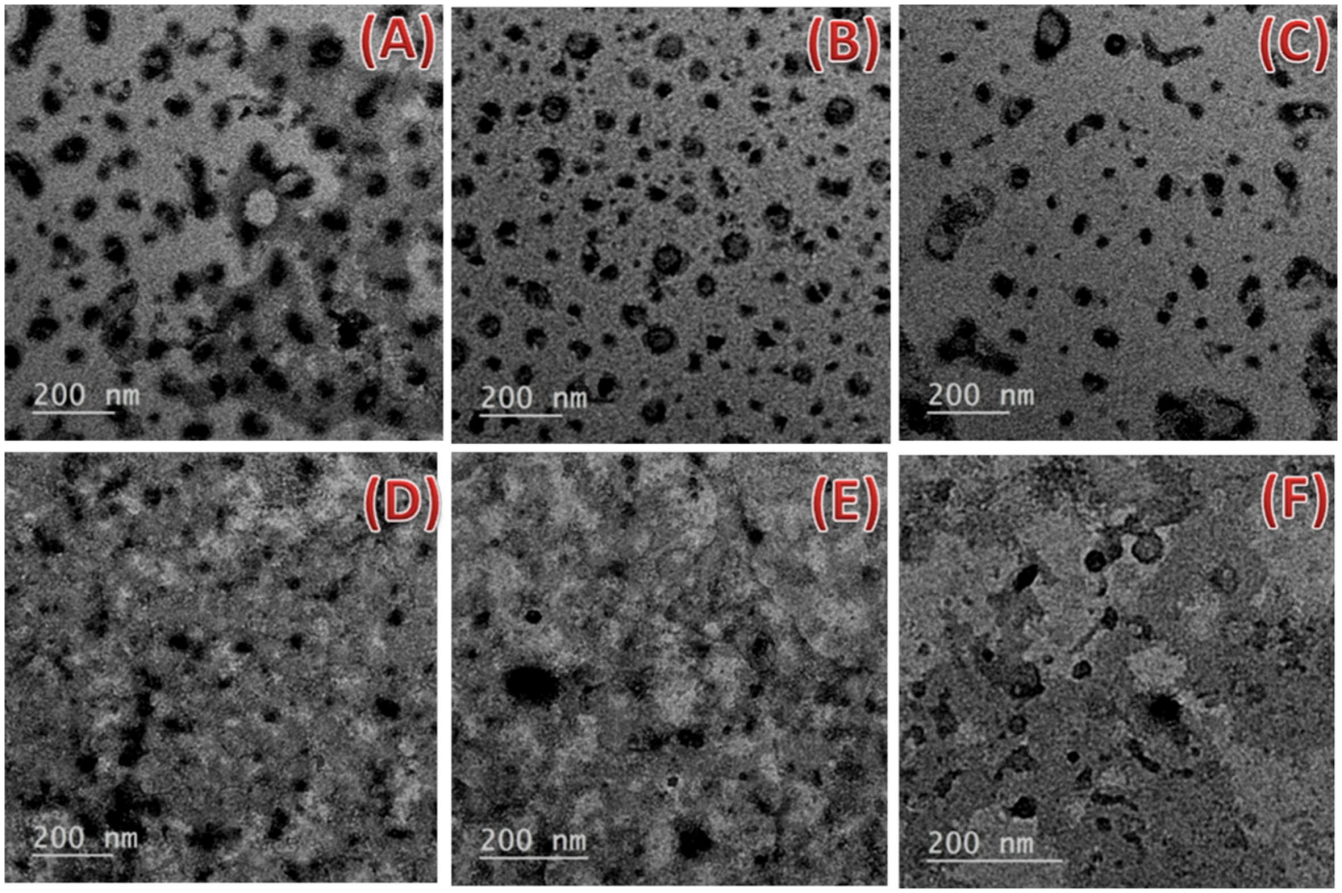
Transmission electron microscopy micrographs of the nanoemulsions prepared with the *Nigella sativa* oil using two different surfactants **(A–C)** 0_T20, 5_T20, and 10_T20 and **(D–F)** 0_T80, 5_T80, and 10_T80 at 200 nm scale bar.

The transmission electron microscope (TEM) images for freshly prepared nanoemulsions with two surfactant types (Tween 20 and 80) are shown in Figure 6. It was revealed that each formulation had a varied size distribution and almost spherical droplet shape. Consequently, the formation of distortions as a result of the nanoemulsions’ negative staining and the high vacuum used during operation may change the actual size of the droplets. In TEM photographs, the droplet had an oval and spherical shape. The drying procedure used to prepare the sample could be to cause of the shape change. Droplets of the 10_T20 and 10_T80 nanoemulsion were well dispersed, as can be seen in Figures 6C,F. This may be attributed to the duration of the sonication process. Analogous results by Mohammed et al. (2020) demonstrated that *N. sativa* oil nanoemulsions prepared with various surfactant types have spherical with slight variations. Similar outcomes were also reported by Yuan et al. (2008) for a sunflower oil nanoemulsion that showed spherical shapes of various diameters and was produced with various Tween-20 emulsifier concentrations. In the current study, nanoemulsions made with Tween 20 had smaller particle sizes but also a tendency to assemble more readily than those made with Tween 80, which had bigger particle sizes but strong interfacial layers to prevent droplet aggregation.

### 3.4. Identification of *Penicillium verrucosum*

As mentioned in the material and methods section, *P. verrucosum* was first identified at the morphological level. In this regard, *P. verrucosum* has shown good growth on Yeast Extract Sucrose agar medium (YES), Potato Dextrose Agar (PDA), and Malt Extract Agar medium (MEA), with average colony diameters (25.20 ± 5.14) mm for MEA; (24.42 ± 5.34) mm for PDA and (21 ± 8.22) mm for YES agar after 7 day incubation at 25°C ± 2. On both MEA and PDA, colonies were velutinous to floccose texture, green with a white edge hem and corrugation. Clear copious exudate droplets were present. The reverse colony color was light yellow-brown on MEA and cream yellow on PDA. Velutinous green colonies with a white edge hem and beige brown to terracotta reverse color were typical for *P. verrucosum* grown on YES medium. The fungal Conidiophores on all cultivated media were terverticillate with rough walled-stripe, and phialides were cylindrical tapering to a distinct column. Conidia were smooth-walled or rough-walled, globose to subglobose. Then the identification was confirmed at the molecular level. PCR analyses of genomic *P. verrucosum* were confirmed using uni-F/uni-R primer pair given DNA products with sizes around 670 bp, which confirmed their identity as *P. verrucosum* strains. Amplification of the ITS region with primers ITS1/ITS4 yielded 512–530 bp DNA products. BLASTn analysis of the sequence of *P. verrucosum* amplification product exhibited e-values of zero and 99.12% similarity with GenBank *P. verrucosum* accessions no. KY347870.1, which confirmed morphological characterization. The partial sequences of the ITS regions from *P. verrucosum* isolate were deposited in GenBank with accession no. OP715869.1.

### 3.5. *In vitro* antifungal activity of nanoemulsions against *Penicillium verrucosum*

The antifungal activity of six prepared nanoemulsions was tested at different concentrations (25, 50, and 100%) against *P. verrucosum in vitro*. The percentage of inhibition in the fungal mycelial growth rate was recorded at 4, 6, and 8 days as demonstrated in Table 1. Variable results of tested nanoemulsions on *P. verrucosum*-growth were observed. The results showed that complete inhibition of *P. verrucosum*-growth was obtained by 10_T80 and 10_T20 nanoemulsions at 100% concentration. Also, after 8 days, 5_T20, 5_T80, 0_T20, and 0_T80 reduced the fungal growth by almost 98, 96, 96, and 93%, respectively as compared to the control (Table 1). This unique antifungal activity results are in agreement with the recently published data in Ziedan et al. (2022), indicating the promising antifungal properties of nanoemulsion formulation of clove and black seed oils in controlling the phytopathogenic fungus *Botrytis cinerea,* the causal agent of gray mold on cucumber plants in compared to the chemical fungicide (Topsin M-70; Youssef et al., 2018; Yousef et al., 2022).

**Table 1:** The antifungal activity of the six different nanoemulsions at three different concentrations against *Penicillium verrucosum* in compared to untreated control after 4, 6, and 8 days of incubation at 25°C ± 1.

Nanoemulsions	Concentration (%)	Inhibition rate (%)		
		4 days	6 days	8 days
0_T80	25	30.12 ± 0.5d	35.35 ± 1.2d	43.0 ± 1.2d
	50	80.40 ± 1.3e	82.15 ± 1.2f	91.21 ± 1.4f
	100	78.70 ± 1.2e	90.153 ± 2.8f	93.0 ± 2.1f
	Control	0.0 ± 0.0a	0.0 ± 0.0a	0.0 ± 0.0a
5_T80	25	32.55 ± 0.5d	38.35 ± 2.8d	46 ± 2.5e
	50	81.35 ± 1.3f	85.15 ± 1.09	95.23 ± 1.35 g
	100	88.70 ± 1.1 g	92.153 ± 1.09f	96.0 ± 0.0 g
	Control	0.0 ± 0.0a	0.0 ± 0.0a	0.0 ± 0.0a
10_T80	25	25.55 ± 0.5d	32.35 ± 2.8d	40 ± 2.5d
	50	80.70 ± 1.1f	84.13 ± 1.22f	99.5 ± 0.0 h
	100	82.70 ± 1.1 g	86.153 ± 1.09f	100.0 ± 0.0 h
	Control	0.0 ± 0.0a	0.0 ± 0.0a	0.0 ± 0.0a
0_T20	25	28.55 ± 0.4d	38.35 ± 2.8d	40.3 ± 1.2d
	50	69.35 ± 2.24e	75.15 ± 1.09e	81.23 ± 1.4e
	100	79.70 ± 1.1e	81.153 ± 1.20f	96.15 ± 0.0 g
	Control	0.0 ± 0.0a	0.0 ± 0.0a	0.0 ± 0.0a
5_T20	25	32.55 ± 0.5d	38.35 ± 2.4d	46.42 ± 2.5e
	50	60.35 ± 2.24e	66.15 ± 1.2e	75.23 ± 1.33e
	100	70.70 ± 1.1e	79.15 ± 1.09e	98.21 ± 0.0 h
	Control	0.0 ± 0.0a	0.0 ± 0.0a	0.0 ± 0.0a
10_T20	25	32.55 ± 0.5d	38.35 ± 2.8d	48.2 ± 2.5e
	50	81.22 ± 2.17f	85.15 ± 1.21f	95.23 ± 1.35 g
	100	88.72 ± 2.1 g	95.15 ± 1.24 g	100.0 ± 0.0 h
	Control	0.0 ± 0.0a	0.0 ± 0.0a	0.0 ± 0.0a
*N. sativa* oil	100% purified	7.22 ± 2.8c	10.2 ± 2.5c	12.05 ± 0.5c
Tween 20	1.07 g/ml	1.0 ± 0.5b	1.0 ± 0.0b	1.0 ± 0.0b
Tween 80	1.07 g/ml	1.2 ± 0.0b	1.5 ± 0.0b	1.6 ± 0.0b

Mean ± SD. Values marked with the same letters, within each column, are not statistically different according to *post hoc* test DMRT at *p* ≤ 0.05.

### 3.6. Effects of nanoemulsions on

#### 3.6.1. Seed germination and maize growth

**Figure 7:**
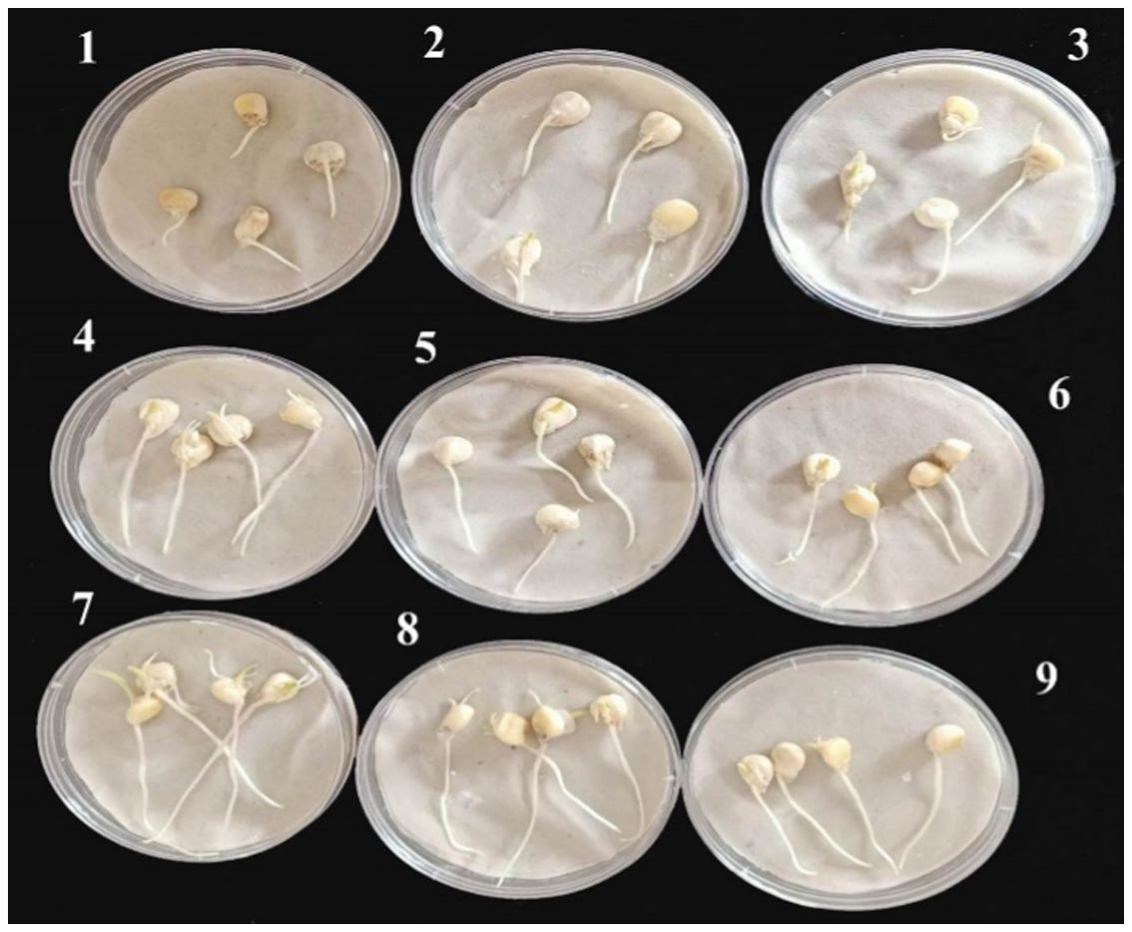
Representative maize seeds at 4 days of germination after their treatment with the six tested nanoemulsions in compared to controls: (1) infected seeds without treatment (+ control), (2) uninfected seeds without treatment (− control), (3) infected seeds with the chemical fungicide, and (4–9) infected seeds treated with (0_T80, 5_T80, 10_T80, 0_T20, 5_T20, and 10_T20) nanoemulsions, respectively.

**Figure 8:**
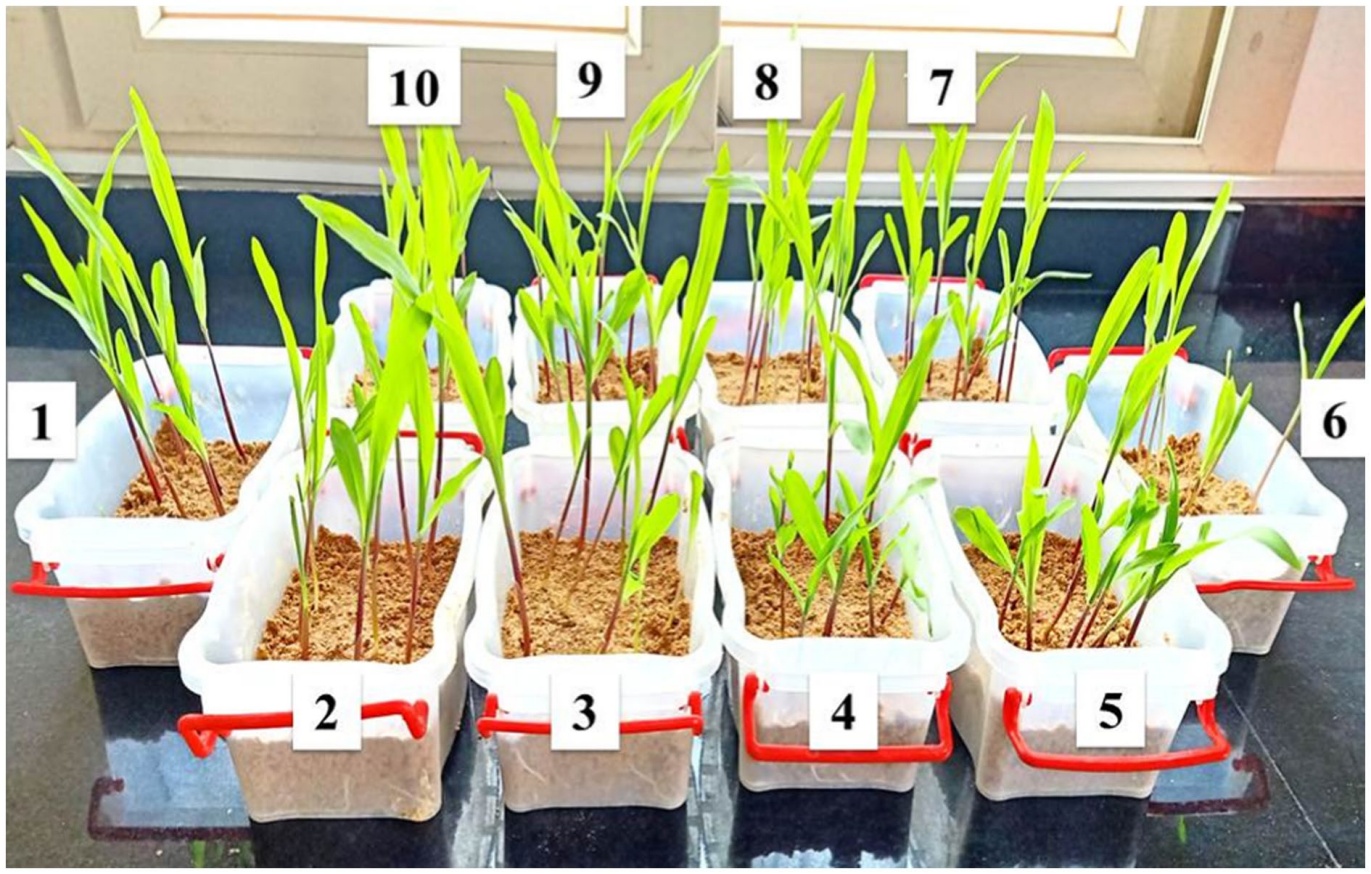
Representative infected samples at 14 days after treatment with the produced nanoemulsions in compared to the chemical fungicide used (1) non-treated control plants (non-infected); (6) non-treated control plants (infected); (5) *Nigella Sativa* oil; (7) fungicide; (2–4) 10_T80, 5_T80, and 0_T80 respectively; and (8–10) 0_T20, 5_T20, and 10_T20, respectively.

Based on data recovered from the *in vitro* antifungal activity of the produced nanoemulsions, nanoemulsions at 100% were selected for further studies. The effect of the six produced nanoemulsions at 100% concentration on maize germination was evaluated and compared to the commercial chemical fungicide “Ridomil Gold SL (active ingredient: Mefenoxam 45.3%) at 2 g/land both control (infected and untreated) seeds (Figure 7; Table 2). Based on data presented in Table 2, all nanoemulsions had increment effects on maize seed germination by 101% in the case of 10_ T20 and 10_ T80 compared to untreated seeds (control) or the chemical fungicide treatment. The percentage of seed germination ranged between 97 and 98.7% and the radical length ranged between 2.2 and 3.9 cm for the six tested nanoemulsions. In particular, the highest germination rate was achieved by 10_T80 and 10_T20 with a rate of 98.7 and 98.2%, respectively. Also, the highest radical length was achieved by 10_T80 and 10_T20 with 3.8 and 3.9 cm, respectively. Overall, the infected seeds showed the lowest germination percentage and radical length (Table 2). In addition, a pictorial representation of the seedling growth upon nanoemulsion treatment were shown in Figure 8. The positive action of those nanoemulsions may be related to that some natural compounds have the potential to stimulate metabolic processes leading to improved seed development (Savy et al., 2017). This effect may be also attributed to a hormone-like action that *Nigella Sativa*-derived oils, and the phenolic compounds it contains, can exert on the early seed’s biochemical activities (R Core Team, 2019). Furthermore, natural-based compounds including essential oils can show a gibberellin-like action that results in a beneficial effect, thus influencing the seed’s hormonal status and physiological mechanisms underlying its development (Savy et al., 2017).

**Table 2:** Effect of the six nanoemulsions treatments on germination of maize seeds and their radical length after infection with *P. verrucosum*.

Treatments	Germination (%)	Radical length (cm)
0_T80	97.0c	2.62b
5_T80	97.7b	2.21c
10_T80	98.7a	3.8a
0_T20	97.7b	3.25a
5_T20	98.2a	3.92a
10_T20	98.2a	3.92a
Chemical fungicide	97.0c	1.75d
Tween 80 (T_80)	97.5b	2.4ab
Tween 20 (T_20)	97.5b	2.3ab
*N. sativa* oil	97.5b	1.8d
Untreated control seeds (non-infected)	97.5b	2.4ab
Untreated control seeds Infected plants (Infected)	65.0d	1.0e

The germination rate and radical length were recorded 4 and 5 days after the treatments, respectively. Values marked with the same letters, within each column, are not statistically different according to *post hoc* test DMRT at *p* ≤ 0.05.

#### 3.6.2. Root and shoot length

**Figure 9:**
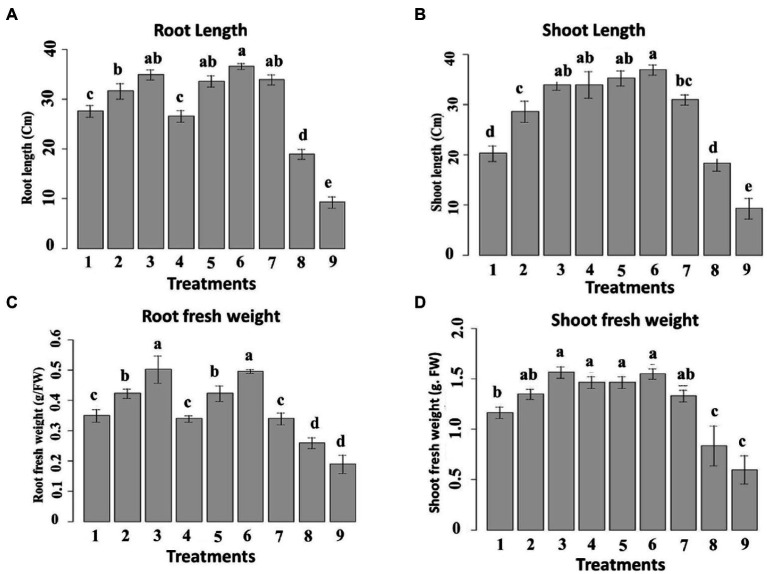
Effect of six nanoemulsions treatments on **(A)** the root and **(B)** shoot length, **(C)** root fresh weight, and **(D)** shoot fresh weight of maize samples: 1–6 are 0_T80, 5_T80, 10_T80, 0_T20, 5_T20, and 10_T20, respectively. 7–9 are chemical fungicide, untreated control, and infected control, respectively. The values were recorded on seedlings at 14 DPS. Values marked with the same letters are not statistically different according to *post hoc* test DMRT at *p* ≤ 0.05.

The prepared nanoemulsions differently affected shoot and root length in maize (Figure 9) terms of both shoot and root length, it was noticed that the applied nanoemulsions treatments “particularly (10_T20 and 10_T80) were able to more stimulate both root and shoot length as compared to the chemical fungicide treatment. Continually, the different treatment solutions on the shoot and root fresh weight in maize were monitored (Figure 9). The obtained results were also in parallel with root and shoot length data, where all nanoemulsions treatments did not passively affect root and shoot fresh weight parameters and both T0_T20 and 10_T80 nanoemulsions showed the highest values compared to other nanoemulsions. In this regard, it has been hypothesized that organic-based compounds including oil-based compounds could increase seed germination of its capacity to act directly on mitotic activity (Popa et al., 2008). Also, the efficacy of seed priming can impact the plant’s subsequent development and also inhibit/reduce the infection with *P. verrucosum* and consequently reduce the mycotoxins secreted by this fungal pathogen, therefore these favorable effects on the earlier growth and maturation are important and investigated. In this regard, 2 weeks after seeding, the maize seedlings were measured for root, shoot lengths, and weights for all treatments (Figure 9). The obtained data are in agreement with other studies indicating that plant-derived products are effective against different fungal pathogens infecting maize plants such as *F*. *verticillioides*, *Colletotrichum graminicola*, and *Fusarium solani* (Abo-Elyousr et al., 2009; Mandal et al., 2009; Masum et al., 2009).

#### 3.6.3. Chlorophyll, carotenoid, anthocyanin, and soluble protein content

**Figure 10:**
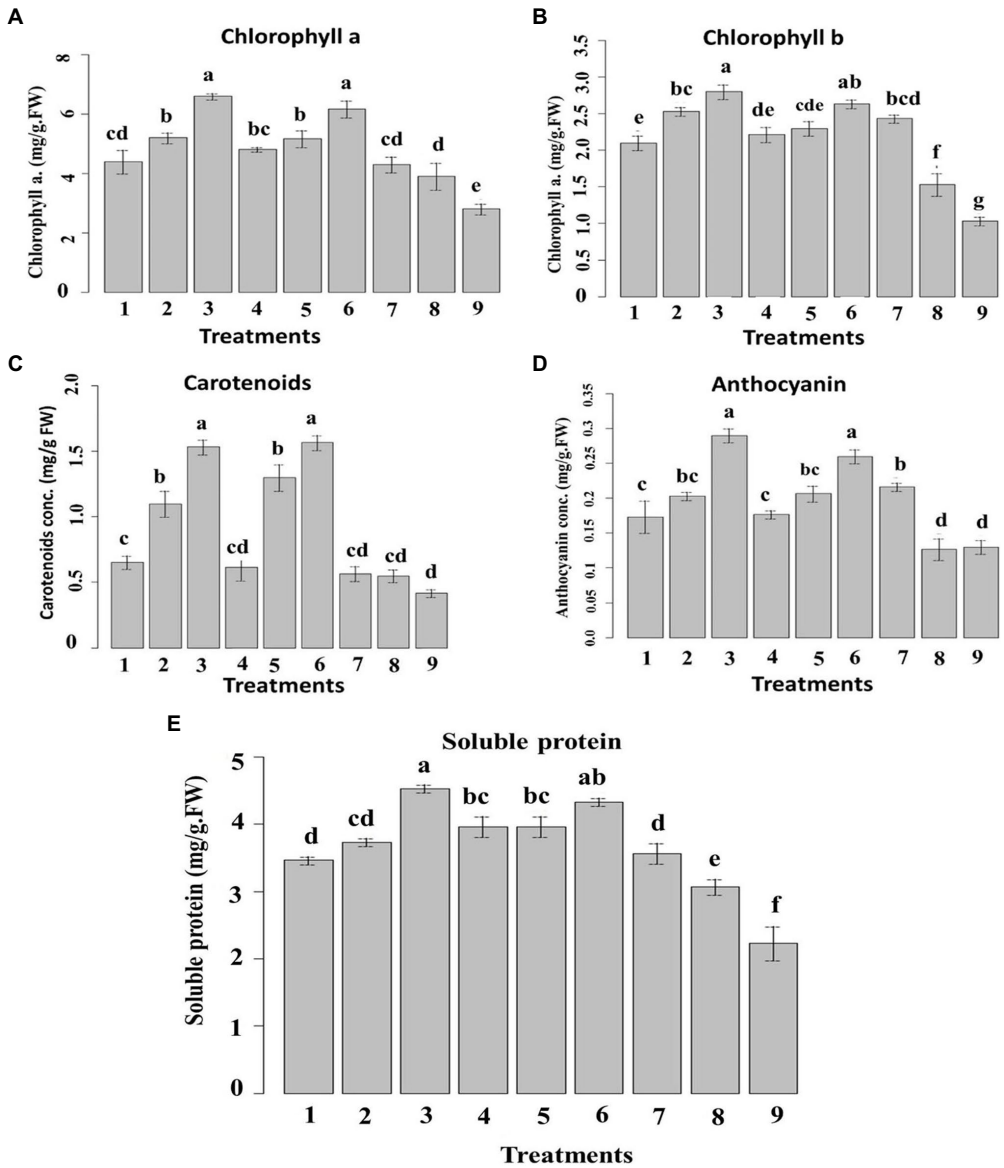
Effect of six nanoemulsions treatments on **(A)** chlorophyll a, **(B)** chlorophyll b, **(C)** carotenoid, **(D)** anthocyanin, and **(E)** soluble protein content in maize leaves compared to the chemical fungicide and (positive and negative) controls. 1–6 are 0_T80, 5_T80, 10_T80, 0_T20, 5_T20, and 10_T20, respectively. 7–9 are chemical fungicide, untreated control, and infected control, respectively. Values marked with the same letters are not statistically different according to *post hoc* test DMRT at *p* ≤ 0.05.

The chlorophyll a and b content was ascertained in the samples exposed to the prepared nanoemulsions (Figure 10). 10_T80 and 10_T20 nanoemulsions showed the highest chlorophyll content followed by 5_T80 and 5_T20. Infected control showed the lowest chlorophyll content (Figure 10A). Concerning chlorophyll b content, 10_T80, 0_T20 and 5_T80 nanoemulsions provoked the highest increases as compared to the chemical fungicide and control samples (Figure 10B). The inductive effects of nanoemulsions on chlorophyll a and b are due to a stimulatory influence on their production. Because of their ability to promote plant nutrition, particularly nitrogen absorption, several phenolic compounds can increase the concentration of the pigments described above (Tanase et al., 2014).

Regarding carotenoids, Figure 10C showed that samples treated with 10_T80, and 10_T20 nanoemulsions demonstrated the highest increases in carotenoids content followed by 5_T80 and 5_T20 as compared to other treatments. Concerning anthocyanin, the treatments with 10_T80 and 10_T20 nanoemulsions showed the highest substantial increases in its content in comparison to other treatments. Also, 5_T80, 5_T20, and chemical fungicide treatment demonstrated no significant differences in anthocyanin content (Figure 10D). As for soluble protein, 10_T80 and 10_T20 nanoemulsions demonstrated the highest increase in soluble protein content followed by 0_T20, 5_T20, and 5_T80 with no significant difference between them (Figure 10E).

The rise in carotenoids seen can be attributed to a protective response to nanoemulsion therapy. The concentration of chlorophyll is also correlated to the number of carotenoids involved. Increases in the level of this pigment are perceived by plants as a signal, which drives carotenoid production (Zoufan et al., 2020). Based on these findings, using non-excessive dosages of nanoemulsions as a way to increase the synthesis of these beneficial compounds is recommended. In terms of anthocyanin, increases were seen in maize following various nanoemulsion treatments. Anthocyanin is a flavonoid that has important functions in plants as well as potential health benefits in humans (Bu et al., 2020). Anthocyanin can prevent and contrast lipid peroxidation by acting on ROS in the vacuole, and their content can be elevated in response to diverse environmental conditions (Dobrikova et al., 2021).

The total soluble protein content, as this is significantly backed by the ability of the plant to assimilate nutrients, can also ensure phytotoxicity (Venkatachalam et al., 2017; Alzahrani and Rady, 2019). The favorable impact of natural oils “nanoemulsions” on maize seedlings might explain this good effect. As previously stated, they can increase in treated plants the nitrogen content, which is crucial for protein biosynthesis (Ertani et al., 2011). In agreement with the above-mentioned data, different reports indicated that plant metabolites can induce systemic resistance in host plants, disease reduction, and increased different plant growth parameters in many crops (Nandakumar et al., 2001; Ramamoorthy et al., 2002). When activated by different biotic or abiotic factors, plant defense genes that are quiescent in healthy uninoculated plants can induce systemic resistance against disease (Naz et al., 2021). Furthermore, it should be noted that a good root development, as prompted by all nanoemulsion treatments, can justify the highest content of chlorophyll a and b since a better root system can allow maize to increase its capacity to take up nutrient from the growth media (Lucini et al., 2018). The amount of carotenoids is also related to the content of chlorophyll a. Indeed, plants can perceive increases in the content of this pigment as a signal which, in turn, stimulates the carotenoid biosynthesis (Bartucca et al., 2020). Based on these results, the use of non-excessive doses of nanoemulsions particularly 10_T80 and 10_T20 can be proposed as a strategy not only for disease reduction by also to raise the production of these beneficial substances.

#### 3.6.4. Activity of the defense-related enzyme and pathogenesis-related proteins in maize leaves

**Figure 11:**
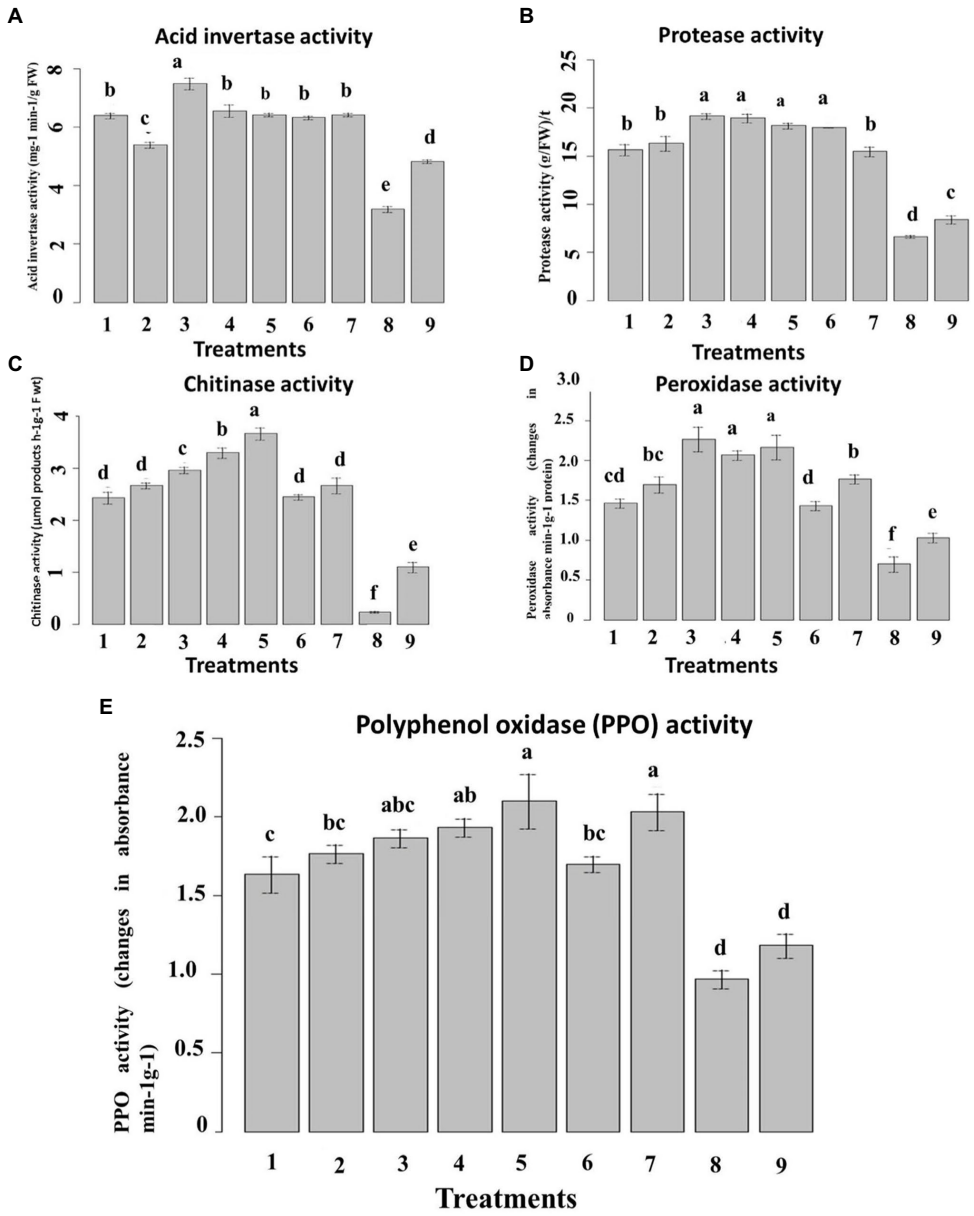
Effect of six nanoemulsions treatments on defense-related enzyme and pathogenesis-related proteins in maize leaves: **(A)** acid invertase activity (IA), **(B)** protease activity, **(C)** chitinase activity, **(D)** peroxidase activity, and **(E)** polyphenol oxidase (PPO): 1–6 are 0_T80, 5_T80, 10_T80, 0_T20, 5_T20, and 10_T20, respectively. 7–9 are chemical fungicide, untreated control, and infected control, respectively. Values marked with the same letters are not statistically different according to *post hoc* test DMRT at *p* ≤ 0.05.

Inoculation of *P. verrucosum* stimulated the activities of all the tested defense-related enzymes tested in all treatments compared to the un-inoculated control. In the pot experiment, acid invertase (AI) and protease activities in the leaves were higher in inoculated plants compared with the untreated plants (Figure 11). The nanoemulsion 5_T20seed treatment significantly increased the AI and protease activity by 68 and 64%, respectively, compared with both the un-inoculated and the infected controls. Seed treatment with 10_T80 nanoemulsion showed the highest AI and protease activity by 75 and 70%, respectively, as compared to the infected control. No significant differences were found between 0_T80, 10_T80, 0_T20, 10_T20, 5_T20, and chemical fungicide treatments for AI. Also, no significant differences were found between 10_T80, 0_T20, 5_T20, and 10_T20.

In the case of chitinase activity, results indicated that seeds treated with all nanoemulsions increased the chitinase enzyme activity, as compared to the infected control. The highest activity was recorded in seeds treated with 5_T20, 0_T20, and 10_T80 followed by 0_T80, 5_T80, and 10_T20 (Figure 11C). In the same way, inoculation of *P. verrucosum* induced higher PPO and POD activity in the seed-treated plants with all nanoemulsions tested than in untreated controls (Figures 11D,E). In the pot, seed treatment with both 10_T80 and 5_T20 nanoemulsion showed the maximum increase of the leaf POD and PPO activities by 60 and 52%, respectively, as compared with the infected control. Also, 10_T80, 0_T20, and 5_T20 showed the highest POD and PPO activities with no significant differences between them. The results obtained herein summarized that the prepared nanoemulsion treatments had a stimulatory effect on these enzyme activities over and above the effect of the pathogen.

These results reveal the positive effects of exogenous nanoemulsions applications to enhance resistance in maize against *P. verrucosum* infections, which by different reports indicated that exogenous application of specific compounds derived plants can induce resistance in the host plant *via* induction of higher levels of host defense enzymes and Pathogen related (PR)-proteins. This suggests that the produced L-CNPs induce systemic resistance in the host plants, resulting in a reduction in disease development (Paul and Sharma, 2002). Moreover, the onset of induced systemic resistance in plants correlates with the increased activity and expression of PR proteins, such as chitinases, β-1,3-glucanases, acid invertase, and peroxidases; consequently, such proteins are generally used as the key ingredients of systemic acquired resistance (SAR), an inducible immune response of plants that averts further infection or disease spreading to the non-infected parts of the host (Siddaiah et al., 2017; Butt et al., 2019). Different studies reported that compound-derived plants can induce systemic resistance in host plants, disease reduction, and increased plant growth in many crops (Nandakumar et al., 2001; Ramamoorthy et al., 2002).

When activated by various factors, plant defense genes that are quiescent in healthy uninoculated plants can induce systemic resistance against disease. Different reports indicated that the increment levels of chitinase, protease, PPO, POD, and AI enzyme activities (Chen et al., 2000; Nandakumar et al., 2001) as well as expression of β-1,3-glucanase and chitinase genes (Ramamoorthy et al., 2002; Saravanakumar et al., 2007) have been reported to be effective against various fungal diseases.

#### 3.6.5. Scanning electron microscope

**Figure 12:**
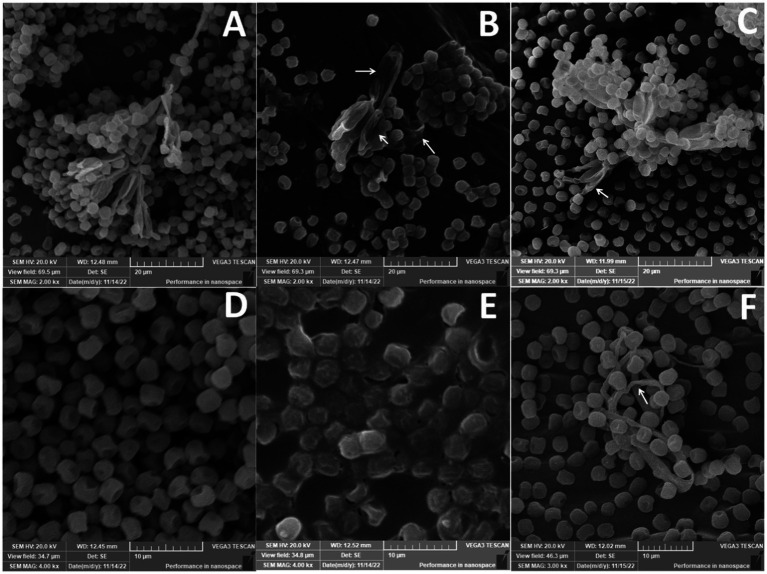
Scanning electron microscope of *Penicillium verrucosum* treated with different nanoemulsions: **(A,D)** Control, **(B,E)** 10_T20, and **(C,F)** 10_T80.

*Penicillium verrucosum* isolate grown on PDA plates showed the characteristic mycelial and conidiospores morphology, with lengthened, normal, and homogenous hyphae of constant diameter with smooth external surfaces and rounded apices (Figures 12A,D). In this regard, SEM micrographs indicated a normal growth for the fungal mycelia of the untreated control (Figures 12A,D), which consisted of conidiophores formed of smooth stipes of 200–500 μm long and ending in typically triverticillate penicillia. The length of the metulae ranged from 10 to 14 μm, while that of the tightly packed phialides was 7–12 μm, Phialide were flask-shaped and more elongated. However, significant morphological changes were noticed in fungal mycelia treated with either 10_T20 or 10_T80 nanoemulsions including alterations in conidiophores, metulae, phialides, and mature conidia characteristics were observed (Figures 12B,C,E,F, arrowed). Substantial morphological changes in the cell wall surface of the fungal mats were also observed and conidiophore improvement was abnormal (Figure 12B, arrowed), where mycelia and conidiophores were shriveled compared to the untreated control. The fungal spores were deformed and collapsed with damaged vesicles (Figure 12E), in compared to the control, and the cell walls became thinner, shriveled, crinkled, and showed a decreased cytoplasmic content and modifications of the membrane integrity, and the fungal conidiophore started to shed its spores (Figures 12C,F). The interactions between nanoemulsion molecules and the polyanionic structure of microbial cell membranes undoubtedly lead to the destabilization of the cell membranes, which is how nanoemulsions affect numerous fungal infections. This causes the fungal pathogen’s intracellular material to flow out, destroying the organism. The primary and secondary mechanisms of antimicrobial activity of nanoemulsions are likely interference with protein synthesis and membrane instability, respectively. The absorption of nanoemulsions into the cellular DNA binding site, which further inhibits RNA and protein synthesis, may also be part of the process by which such nanoemulsions work. Our findings are also in agreement with (Junior et al., 2012), indicating that chitosan nanoparticles may also cause fungal mat accumulation, and structural changes, such as excessive branching, cell wall swelling, and reduced hyphal size, which have been observed in *P. expansum* and *Rhizopus stolonifer.*

## 4. Conclusion

*Nigella Sativa* nanoemulsions were prepared using homogenization followed by ultrasonication process based on two types of surfactants (Tween 20 and Tween 80) and different sonication times. 10_T20 and 10_T80 nanoemulsions showed the smallest particle size and good stability. Six nanoemulsions were used to prime maize seeds, and the results included fascinating biochemical and physiological responses as well as a coating that protected maize seeds from the pathogenic fungus *P. verrucosum*. Nanoemulsions were applied at different concentrations using nano-priming technology. Nanoemulsions namely 10_T20 and 10_T80 had the highest anti-*P. verrucosum* activity, maize seed germination, more stimulate root and shoot length and the highest chlorophyll and carotenoids content. The beneficial effects recorded on maize at certain concentrations offer encouraging prospects. Thus, given the chance provided by the prepared nanoemulsions, more study is required to comprehend the nature of their beneficial effects. The abundance of this biomass, the potential environmental impact of its disposal, and the significance of developing new protective seed coatings and biostimulants for agriculture make this topic of particular interest.

## Data availability statement

The raw data supporting the conclusions of this article will be made available by the authors, without undue reservation.

## Author contributions

MM and AH conceived the research idea and helped to collect the data. MM, KY, SH, and AH analyzed the data and wrote the manuscript. All authors contributed to the article and approved the submitted version.

## Conflict of interest

The authors declare that the research was conducted in the absence of any commercial or financial relationships that could be construed as a potential conflict of interest.

## Publisher’s note

All claims expressed in this article are solely those of the authors and do not necessarily represent those of their affiliated organizations, or those of the publisher, the editors and the reviewers. Any product that may be evaluated in this article, or claim that may be made by its manufacturer, is not guaranteed or endorsed by the publisher.
